# Factors associated with long-acting reversible contraceptives usage among sexually active adolescent girls and young women in Zimbabwe

**DOI:** 10.1371/journal.pgph.0003551

**Published:** 2024-08-20

**Authors:** Isaac Chipako, Saurabh Singhal, Bruce Hollingsworth

**Affiliations:** 1 Health Economics and Policy Division of Health Research Graduate College, Lancaster University, Lancaster, United Kingdom; 2 Economics Department, Lancaster University, Lancaster, United Kingdom; University of Dhaka, BANGLADESH

## Abstract

Despite the benefits of long-acting reversible contraceptives (LARCs), they are not being utilized in Zimbabwe as much as the short-acting reversible contraceptives (SARCs). The aim of the study was to explore factors associated with LARC usage among Zimbabwean adolescent girls and young women, using data from the 2015 Zimbabwe Demographic and Health Survey. Cross tabulations and chi-square tests were used initially to describe associations. Odd ratios were then used to measure the strength of association between LARCs usage and the independent variables using stepwise multinomial logistic regression. From the 2132 sexually active females included in the study 9.1% were LARCs users and 42% were SARCs users at the time of the survey. Secondary and primary education had increased odds of not using any method (OR: 5.032, 95% CI: 2.136–11.8512 and OR: 5.799, 95% CI: 2.327–14.453 respectively) compared to tertiary education. Women with no living children had increased odds of not using any method (OR 66.543, 95% CI: 25.784–171.7381). Being not married was associated with decreased odds of SARCs usage (OR 0.399, 95% CI: 0.285–0.558). Desire for no more children was associated with reduced odds of SARCs usage (OR: 0.448, 95% CI: 0.304–0.66). Being a member of the Apostolic Faith church was associated with increased odds of not using any method (OR 1.423517, 95% CI: 1.018–1.990309). In conclusion, acquiring a tertiary education, having children, a desire to cease bearing children altogether, being unmarried and belonging to the highest wealth class were generally associated with an increased likelihood of using LARCs. Being a member of the Apostolic Faith church was associated with a decreased likelihood of LARCs usage. Findings from the study are relevant in the Zimbabwean context and highlights the relevant factors essential to focus on, when carrying out interventions aimed at increasing LARCs uptake in the country.

## 1. Introduction

Enhancing the utilization of contraception is a cost-efficient and beneficial investment in reducing a high maternal mortality rate and enhancing the well-being and health of women, which in turn benefits the overall welfare of a country [1]. It is widely accepted that modern contraceptive methods reduce the risks of unplanned pregnancies, unsafe abortions, maternal and infant illness and death, and offer various important socio-economic advantages [2–4]. Use of modern contraceptive methods enables individuals to achieve their desired family size and regulate the timing between pregnancies [5].

Modern contraceptive methods consist of user dependent short-acting reversible contraceptives (SARCs) such as oral contraceptives and injectables that work for up to three months. This contrasts with long-acting reversible contraceptives (LARCs) which are less user dependent, and permanent contraceptive methods (vasectomy and tubal ligation) [2, 6, 7]. There are two types of LARC methods: implants (Jadelle, Sino-Implant II and Implanon) and the intrauterine contraceptive devices (IUCDs). Implants are tiny plastic rods that are typically inserted beneath a woman’s skin, often placed on the inner area of the upper arm that function by slowly releasing the hormone progestogen into the woman’s body. Jadelle, Sino-Implant II and Implanon are highly effective (98.8% effective) in preventing pregnancy for up to five, four and three years respectively [6–8]. Whilst the IUCD, commonly referred to as the ‘Loop’, is a safe and extremely effective (99% effective) small, flexible contraceptive device placed inside the uterus to prevent pregnancy [6, 7].

Although SARCs are most used in sub-Saharan Africa (SSA), LARCs are more efficient under “typical use” scenarios, as they remove the requirement for strict adherence and exhibit higher rates of continuation [2]. The use of LARCs can decrease discontinuation, which is frequent among adolescent girls and young women, as they offer long-term benefits and present fewer challenges in terms of adherence [9].

Shoupe and colleagues provided evidence that increased LARCs uptake drastically reduced unplanned pregnancies and induced abortions in the USA [10]. Furthermore, the Johns Hopkins Bloomberg School of Public Health/ and the World Health Organization (WHO) states that LARC methods are safe and suitable for nearly all women respectively. Most women, including multiparous and nulliparous women, married and unmarried women, adolescent girls and women over 40 years, those who have just had an abortion or ectopic pregnancy or miscarriage (with no evidence of infection in the case of IUCDs), breastfeeding women, and women with anemia, are recommended to be able to safely and effectively use LARC methods [8, 11]. Moreover, women who smoke cigarettes, regardless of age or smoking frequency, as well as women living with HIV, can utilize implants. Similarly, women who have had pelvic inflammatory disease can use IUCD devices [12]. Despite significant evidence of benefits associated with LARC methods usage [13] and their recommendation by the WHO, they remain underutilized especially among adolescent girls and young women [2, 9]. Utilizing pooled DHS data from 26 sub-Saharan African countries between 2010 and 2019, Bolarinwa and colleagues reported that 81.80% of sexually active adolescents and young women were not using LARCS [14].

Using data obtained from the Multiple Indicator Cluster Surveys (MICSs) across 20 African countries collected between 2013–2018 Apanga and colleagues reported high utilization of SARC methods with over 74% utilization compared to 16% implants utilization among women of reproductive age (15–49 years) [15]. These findings were validated by Boadu, 2022 who reported similar results using data from the Demographic and Health Surveys (DHSs) conducted between 1995 and 2020 across 37 SSA countries. Boadu, 2022 reported high utilization of SARC methods with over 72.6% utilization compared to 19.3% LARC methods utilization among women of reproductive age [16]. Apanga and colleagues further highlighted high utilization (60%, 95% CI: 58% to 62%) of oral pills in Zimbabwe [16]. Adedini and colleagues (2019) using the Zimbabwe DHS reported a low LARCs uptake of 8.51% among women of reproductive ages [17].

The underutilization of LARCs may be due to misperceptions about their safety because of initial design flaws, issues with insertion and removal, knowledge and misperceptions about effects on future fertility [17, 18]. Despite recent evidence suggesting that currently available LARCs methods are safe, user-friendly, highly effective, have long lasting effects, and are easily reversible with rapidly restored fertility upon reversal [17], the rate of LARCs usage remains low among adolescent girls and young people (17). To increase LARCs utilization in Zimbabwe, the Ministry of Health and Childcare received funding to enhance the availability of LARCs nationwide as part of a population-wide family planning initiative. Additionally, the adolescent sexual and reproductive health (ASRH) strategies being implemented in Zimbabwe may improve LARC methods utilization though equal access and knowledge to LARCs between adolescent girls and young women [19]. However, to the best of our knowledge no analysis had been done to determine the factors associated with the use of LARC methods among adolescent girls and young people in Zimbabwe when this study was conducted.

Nonetheless, extensive research has been undertaken to understand factors influencing the uptake of modern contraceptives among women of reproductive ages [20]. Socioeconomic, demographic and sexual characteristics factors have been reported to be associated with the use of modern contraceptives among women of reproductive age in several SSA countries [17, 15, 16, 21] including Zimbabwe. Women who require contraception may choose not to utilize a method for various reasons, such as limited geographic or financial access, health worries or side effects, and limited decision-making authority [22].

In Zimbabwe adolescents and young people aged 15–24 years constitute 20% of the population. Furthermore, 42% of women of reproductive ages, and 34% of maternal deaths in Zimbabwe are within this age-group [23]. The national assessment report on adolescent pregnancies in Zimbabwe conducted in 2023 indicated that the prevalence of adolescent pregnancies was 23.7% among adolescents aged 10 to 19 years. The report also noted that 0.9% of girls aged 10-14-year-olds and 41.2% of 15-19-year-olds were pregnant [24]. These statistics highlight the significant issue of unintended pregnancies among young people in Zimbabwe, underscoring the need for targeted reproductive health interventions and education. Compounded with the fact that LARC methods are not being utilized as much as the pill in SSA countries, exploring factors that influence contraceptive use may guide interventions aimed at increasing LARCs awareness and promote their use among adolescent girls and young women [3]. Henceforth, the present study’s aim was to investigate further the influence of socioeconomic and demographic factors such as type of residence, highest education level, age, economic status, religion, attitude towards fertility, parenthood, and marital status on LARCs usage among Zimbabwean adolescent girls and young women.

## 2. Methods

### 2.1 Ethical statement

There was no requirement for ethical approval because the study was carried out using secondary published Zimbabwe Demographic Household Survey (ZDHS) datasets, without direct human beings’ involvement. The study was conducted under the original consent provided by participants in the ZDHS. Formal approval to access and use ZDHS datasets was granted to Isaac Chipako by the Demographic and Health Surveys (DHS) programme.

### 2.2 Data sources

Data from the ZDHS 2015 was utilized for the study. The ZDHS is a survey conducted every five years by the Government of Zimbabwe, capturing data on population and health indicators including household characteristics, fertility, and maternal and child health through a cross-sectional approach. The ZDHS in 2015 covered 10534 households, from which a total of 9955 women were interviewed, including 2156 (21.7%) adolescent girls aged 15–19 and 1782 (17.9%) young women aged 20–24. Information was collected from women aged 15–24 through the SPSS individual women recode files (ZWIR72FL.SAV).

### 2.3 Data variables/dictionary

The dependent or outcome variable in the study was current method of contraception (V313), which was recoded as 0 if not using any method, 1 if using any LARC (IUCD or implants/Norplants) and 2 if using any SARC method (male and female condoms, injection, diaphragm, lactational amenorrhea, emergency contraception, pill). Abstinence and withdrawal were considered as traditional methods and were excluded from any further analysis.

The dependent variable was obtained from two ZDHS questions: *‘Are you currently doing something or using any method to delay or avoid getting pregnant*?*’* and *‘Which method are you using*?*’*

Using guidance from models conducted elsewhere in the literature [15, 16, 21], independent variables were socioeconomic and demographic factors selected from ZDHS household- and female-level characteristics (S1 File). The household-level variables were wealth status (V190), and type of residence (classified as urban dwelling or rural dwelling) (V025), while the female-level variables were respondent’s current age (V012), age in 5-year age groups (VO13), highest education level (V106), marital status (V501), religion (V130), number of living children (V218), and desire for children in the future (V605). Some variables were recoded to suit the focus of the study. Religion was recoded as None, Apostolic faith, Pentecostal, Protestant, Roman Catholic, and other (Muslim/Traditional/other Christian/other) (1, 2, 3, 4, 5 and 6 respectively). Marital status was recoded into either being married or not married from the original categories (living with a partner was considered married), desire for more children renamed attitude towards fertility was coded as those who did not want or those that wanted children (Coded 1 and 2 respectively), and the number of living children was renamed living children and categorised as none and yes. Age, residence, wealth index, and education levels were retained in their original categories.

### 2.4 Data analysis

Age was expressed in terms of median and interquartile range (IQR) and 5-year age group frequencies. Descriptive statistical cross-tabulations were performed to analyze the variations in LARCs utilization based on different independent variables. Pearson’s Chi-Squared test (χ2) was utilized to assess the statistical significance of the chosen independent variables in relation to LARCs usage. Relative risk ratios or odd ratios (OR) were employed to assess the strength of the association between LARCs usage and the independent variables. Multinomial logistic regression was utilized to assess the relationship between a group of independent variables and the nominal dependent variable with three groups (LARCs usage, SARCs usage and non-use). Backwards stepwise multinomial logistic regression models were used. Variables that did not demonstrate a significant association with LARCs usage at p = 0.20 level were systematically removed from analysis. The focus of this study was on LARCs usage, making it the reference category in the regression analysis. Except for the stepwise logistic regression which was performed using STATA/Be 17, all data were analysed with IBM SPSS Statistics for Windows, version 19.0 (IBM Corp., Armonk, NY, USA). *p*-values <0.05 were considered significant during the analysis. Bar graphs were drawn with IBM SPSS. According to the DHS Guide to statistics, sample weights were applied only for descriptive statistics (percentages, means) to adjust for over sampling and under sampling using the provided women’s individual sample weight (v005/1000000) [25].

## 3. Results

### 3.1 Socio-economic and demographic characteristics

**Fig 1:**
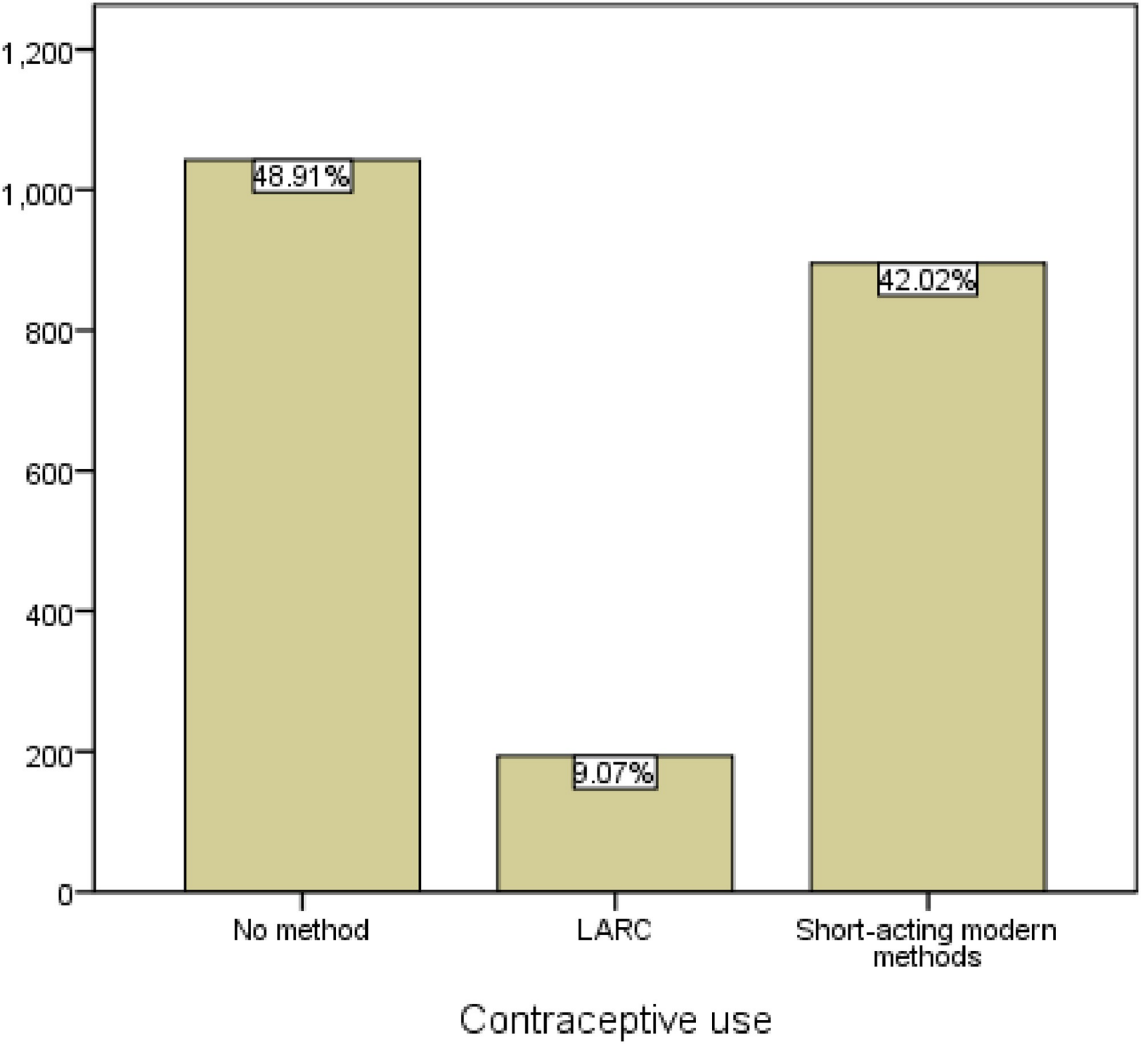
Modern contraception use by sexually active adolescent girls and young women aged 15–24 in ZDHS 2015.

The ZDHS recruited a total of 3938 adolescent girls and young women, with 2156 (54.75%) being adolescent girls aged 15–19 years and 1782 (45.25%) being young women aged 20–24. The DHS data further demonstrates that 2826 (71.76%) of these women reported that they were not using any method of contraception at the time of the survey. Amongst the users, 1092 (27.73%) used modern methods (see S2 File for contraception use distribution) and 10 (0.25%) used traditional methods, 3 (0.08%) used periodic abstinence and 7 (0.18%) used withdrawal). After removal of the non-sexually active group, traditional methods users, infecund women and weighting, the study consisted of 2132 sexually active participants, of which 721 (33.8%) were adolescent girls and 1411 (66.2%) were young women. The median age was 21 (interquartile range 19–23). From the remaining 2132 adolescent girls and young women, 1043 (48.9%) were non-users, 193 (9.1%) were LARCs users and 896 (42%) were SARCs users at the time of the survey, notwithstanding their marital status (Fig 1). The socio-economic demographic characteristics of the adolescent girls and young women are provided in Table 1.

**Table 1:** Socio-economic and demographic characteristics of sexually active adolescent girls and young women aged 15–24 in ZDHS 2015.

Variable		Number	Percentage (%)
Age group in 5 years	15–19	721	33.8
	20–24	1411	66.2
Residence	Urban	684	32.1
	Rural	1448	67.9
Education levels	No education	11	0.5
	Primary education	618	29.0
	Secondary education	1436	67.3
	Higher education	67	3.1
Religion	None	132	6.2
	Apostolic sec	955	44.8
	Pentecostal	543	25.5
	Protestant	277	13.0
	Roman catholic	106	5.0
	Other	119	5.6
Wealth Index	Poorest	418	19.6
	Poorer	440	20.6
	Middle	402	18.9
	Richer	496	23.2
	Richest	377	17.7
Living children	None	611	28.7
	One or more children	1521	71.3
Current marital status	Not married	666	31.3
	Married	1466	68.7
Desire for more children	Desire no more	262	12.3
	Undecided	63	3.0
	Desire more children	1807	84.8

### 3.2 Contraception use among young Zimbabwean women aged 15–24 in 2015

Cross-tabulations were conducted to identify any statistical relationships between the variables under examination and the three categories of non-use, LARCs usage, and SARCs usage. The results are presented in Table 2 and S3 File. Except for education (*p* = 0.642) there were significant associations between the study variables and LARCs usage (*p*<0.05). As expected, non-use was highest across all the independent variables followed by SARCs usage, and LARCs usage was the lowest (see Table 2). LARCs usage was high among young women (20–24) 11.3% compared to adolescent girls (15–19) 4.6%. In the wealth status category richer women had the largest proportion of LARCs usage at 12.3%. In terms of religion those from the Pentecostal church constituted the largest proportion of LARCs usage at 12%. As expected LARCs usage was higher among urban dwelling women 11.5% compared to rural dwelling women 7.9%. Women who did not desire to have more children had the highest proportion of LARCs usage at 20.2%. Likewise, women with children had a higher proportion of LARCs usage (12.5%) compared to women who did not have children (0.5%). Women that were not married had a higher proportion of LARCs usage at 10.9% compared to married women at 8.3%.

**Table 2:** Contraception use in women aged 15–24 in Zimbabwe in 2015.

Variable		Contraceptive use			*p-value*	*χ* ^2^
		None	LARC usage	SARC usage		
Age group in 5 years	15–19	456 (63.2%)	33 (4.6%)	232 (32.2%)	0.000	94.942
	20–24	587 (41.6%)	160 (11.3%)	664 (47.1%)		
Residence	Urban	343 (50.1%)	79 (11.5%)	263 (38.4%)	0.007	10.012
	Rural	700 (48.3%)	115 (7.9%)	633 (43.7%)		
Education levels	No education	4 (33.3%)	1 (8.3%)	7 (58.3%)	0.642	4.257
	Primary education	294 (47.6%)	52 (8.4%)	272 (44.0%)		
	Secondary education	714 (49.8%)	136 (9.5%)	585 (40.8%)		
	Higher education	31 (45.6%)	5 (7.4%)	32 (47.1%)		
Religion	None	47 (35.6%)	15 (11.4%)	70 (53.0%)	0.016	21.886
	Apostolic sec	483 (50.5%)	74 (7.7%)	399 (41.7%)		
	Pentecostal	264 (48.7%)	65 (12.0%)	213 (39.3%)		
	Protestant	139 (50.0%)	17 (6.1%)	122 (43.9%)		
	Roman catholic	55 (51.9%)	10 (9.4%)	41 (38.7%)		
	Other	55 (46.2%)	13 (10.9%)	51 (42.9%)		
Wealth Index	Poorest	196 (46.9%)	29 (6.9%)	193 (46.2%)	0.002	24.681
	Poorer	208 (47.3%)	46 (10.5%)	186 (42.3%)		
	Middle	208 (51.7%)	20 (5.0%)	174 (43.3%)		
	Richer	229 (46.3%)	61 (12.3%)	205 (41.4%)		
	Richest	202 (53.6%)	37 (9.8%)	138 (36.6%)		
Living children	None	539 (88.2%)	3 (0.5%)	69 (11.3%)	0.000	5.327E2
	1 or more	504 (33.1%)	191 (12.5%)	827 (54.3%)		
Current marital status	Not married	438 (65.7%)	73 (10.9%)	156 (23.4%)	0.000	1.395E2
	Married	608 (41.4%)	121 (8.2%)	741 (50.4%)		
Desire for more children	Desire no more	120 (45.8%)	53 (20.2%)	89 (34.0%)	0.000	62.156
	Undecided	43 (68.3%)	8 (12.7%)	12 (19.0%)		
	Desire more children	880 (48.7%)	132 (7.3%)	795 (44.0%)		

*p*-value< 0.05 indicates that there is a statistical relationship between the categorical variables.

### 3.3 Backwards stepwise multinomial logistic regression estimates to determine the factors associated with LARCs

Variables were included in the multinomial regression model, with LARCs usage as the reference category for contraceptive use. Reference categories for the independent variables are indicated for each variable.

#### 3.3.1 LARCs-usage versus non-use

Age, type of residence, current marital status and attitude towards fertility were not statistically associated with LARCs usage among adolescent girls and young women after running backwards stepwise multinomial logistic regression (*p*-values >0.05, see Table 3 for details). Highest education level, parenthood, being a member of the Apostolic Faith church and highest wealth class status were statistically associated with increased LARCs usage among adolescent girls and young women (*p*-values <0.05). Adolescent girls and young women with secondary and primary education had increased odds of not using any method (OR: 5.033, 95% CI: 2.136–11.852 and OR: 5.799, 95% CI: 2.327–14.453 respectively) hence lower odds of using LARCs than adolescent girls and young women who received tertiary education. Women without living children had increased odds of not using any method of contraception (OR 66.543, 95% CI: 25.784–171.738) and decreased odds of LARCs usage than women with living children. Women of the Apostolic Faith church had increased odds of not using any method of contraception (OR 1.424, 95% CI: 1.018–1.990) and decreased odds of LARCs usage. Women in the poorest and middle wealth class status had increased odds of not using any method (OR 1.766, 95% CI: 1.040–2.702 and OR 1.677 95% CI: 1.272–3.512) compared to women in the highest wealth class status.

**Table 3:** Stepwise multinomial logistic regression model estimates for use of LARCs among sexually active females aged 15–24 Zimbabwe, 2015.

Contraceptive use		*p*-value	Odds Ratio (OR)	95% Confidence Interval	
				Lower Bound	Upper Bound
No method	**Attitude towards fertility**				
	Desire no more children	0.570	0.893	0.606	1.317
	Undecided	0.456	1.367	0.601	3.106
	Desire more children	[Ref]	[Ref]	[Ref]	[Ref]
	**Living children**				
	Non	0.000	66.543	25.784	171.738
	Yes	[Ref]	[Ref]	[Ref]	[Ref]
	**Highest education levels**				
	Primary education	0.000	5.799	2.327	14.453
	Secondary education	0.000	5.032	2.13	11.852
	Tertiary education	[Ref]	[Ref]	[Ref]	[Ref]
	**Religion**				
	Apostolic faith	0.039	1.424	1.018	1.990
	No religion	[Ref]	[Ref]	[Ref]	[Ref]
	**Wealth Index**				
	Poorest	0.034	1.677	1.040	2.702
	Middle	0.004	2.114	1.272	3.512
	Richest	[Ref]	[Ref]	[Ref]	[Ref]
	**Current marital status**				
	Not married	0.143	0.775	0.551	1.090
	Married	[Ref]	[Ref]	[Ref]	[Ref]
SARC	**Attitude towards fertility**				
	Desire no more children	0.000	0.448	0.304	0.660
	Undecided	0.086	0.461	0.191	1.115
	Desire more children	[Ref]	[Ref]	[Ref]	[Ref]
	**Living children**				
	Non	0.000	6.443	2.476	16.767
	Yes	[Ref]	[Ref]	[Ref]	[Ref]
	**Education levels**				
	Primary education	0.47	1.347	0.593	3.061
	Secondary education	0.665	1.183	0.552	2.535
	Tertiary education	[Ref]	[Ref]	[Ref]	[Ref]
	**Religion**				
	Apostolic sec	0.897	1.022	0.738	1.414
	No religion	[Ref]	[Ref]	[Ref]	[Ref]
	**Wealth Index**				
	Poorest	0.067	1.545	0.970	2.459
	Middle	0.006	2.024	1.230	3.332
	Richest	[Ref]	[Ref]	[Ref]	[Ref]
	**Current marital status**				
	Not married	0.000	0.399	0.285	0.558
	Married	[Ref]	[Ref]	[Ref]	[Ref]

OR values > 1suggests increased odds or likelihoods

OR values < 1suggests decreased odds or likelihoods

OR with a narrow confidence interval suggests a precise estimate of the association

*p*-value< 0.05 indicates the association is significant

Ref = reference category

#### 3.3.2 LARCs-usage versus SARCs usage

Highest education level, type of residence, age and religion were not statistically associated with LARCs usage among adolescent girls and young women (*p*-values >0.05, see Table 3 for details). Current marital status, wealth index, parenthood and attitude towards fertility were statistically associated with LARCs usage among adolescent girls and young women (*p*-values <0.05). Adolescent girls and young women not married were associated with decreased odds of SARCs usage (OR 0.399, 95% CI: 0.285–0.558) and increased odds of LARCs usage compared to married women.

Adolescent girls and young women who desired no more children had reduced odds of SARCs usage (OR: 0.448, 95% CI: 0.304–0.660) and increased odds of LARCs usage than those who desired to have more children. Women with no children had increased odds of SARCs usage (OR: 6.442, 95% CI, 2.276–16.761) and reduced odds of LARCs usage than women with children. Women in the middle wealth class had increased odds of SARCs usage (OR 2.024, 95% CI: 1.230–3.332).

## 4. Discussion

While SARCs are highly efficacious methods to prevent pregnancy, they result in significantly higher failure rates in typical-use compared to LARCs and these rates are increased among adolescent girls and young women [7, 26]. The predicted 12 months failure rates among all reproductive age women including adolescents with typical-use are 15% for condoms, 8% for oral contraceptive pills, 3% for depo-medroxyprogesterone injectables, while they are less than 1% for LARCs. LARCs also offer non contraceptive benefits such as reducing menstrual blood flow and dysmenorrhea [27]. Additionally, LARCs represent a form of contraceptive that do not contain the hormone estrogen, making them safe for women with contraindications to estrogen-containing medications [18]. Despite the numerous advantages associated with LARCs usage and the WHO recommendations in place many adolescent girls and young women in low-income countries still rely on the more user dependent SARCs [17, 28].

Indeed, low LARCs usage of 9.1% was observed in the present study, compared to SARCs usage of 42% among adolescent girls and young women. This is comparable to the finding by Adedini and colleagues (2019) who reported low LARCs usage (8.51%) among Zimbabwean women of reproductive age [17]. The findings are in agreement with studies conducted in SSA, which reported overall low LARCs usage among women of the reproductive age compared to SARCs usage [15, 16, 21]. Nonetheless, in some SSA countries such as Kenya, the uptake of implants has risen to become second most popular contraceptive method after injections, however use of IUCD still remains low [9]. Hence, this study investigated the influence of socioeconomic and demographic factors on LARCs usage in comparison to SARCs usage and non-use among Zimbabwean adolescent girls and young women.

In contrast to other studies, age and type of residence (urban dwelling vs rural dwelling) which have been previously reported to have significant associations with modern contraceptive usage [7, 9, 17, 29, 30] were not found to be significant predictors of LARCs usage. The non-significant relationship between LARCs usage and type of residence was perhaps an unexpected finding, as there is normally increased access to LARCs health facilities in urban areas versus rural areas [7]. Interventions such the ASRH strategies that aimed to provide equal access and knowledge to SRH issues among young Zimbabweans and ensuring the availability of LARCs nationwide as part of a population-level family planning strategy may have led to equitable access to LARCs in both rural and urban areas.

In the marital status category being married or living with a partner was associated with increased odds of SARCs usage and reduced odds of LARCs usage among the adolescent girls and young women. This was perhaps expected since married adolescent girls and young women may have plans to start a family soon and may therefore be reluctant to use LARCs, which are considered long-term and believed to delay fertility. They may opt for SARCs instead, as they align better with their short-term family planning goals [9, 17].

Women who did not have children had increased odds of not using any method and SARCs usage, and decreased odds of LARCs usage compared to women with children. Several studies from other Sub-Saharan countries have reported similar results [14, 17]. Adedini and colleagues reported that the likelihood of LARCs usage was significantly much higher among multiparous women compared to their childless counterparts [17]. This may be attributed to the fact that women who have had multiple pregnancies are more likely to receive family planning education and counseling on the use of LARCs during their pregnancy, thus enhancing their likelihood of using LARCs [14]. Another possible explanation is that multiparous women could be using LARCs to limit childbearing as they would have attained their desired number of children. Adolescent girls and young women no longer desiring more children had reduced odds of SARCs usage and increased odds of LARCs usage. This would be expected as LARCs are mostly used for limiting childbearing and the results are similar to findings by Adedini and colleagues [17].

When LARCs usage is compared to non-use adolescent girls and young women with secondary and primary education had decreased odds of LARCs usage compared to those with tertiary education. This implies that young women with tertiary education had increased odds of LARCs usage compared to adolescent girls and young women with secondary and primary education. A plausible explanation for this observation could be that highly educated women typically have better access to information regarding the potential side effects and advantages of using LARC methods. Consequently, they are knowledgeable about the misconceptions and myths that commonly act as barriers to the use of LARCs [14]. However, this contradicts findings contradicts from a similar study in Kenya where education was concluded to have no significant influence on LARCs usage. In Kenya, it was argued that better information and exposure to contraceptive services linked to augmented access to education has been overtaken by improved information, better access, and availability for all potential users in the promotion of LARC methods [31]. Therefore, these parameters can also be exploited in Zimbabwe and thereby cancel out the influence of education.

The findings concerning the religion variable when LARCs usage is compared to non-use are not surprising, as the Apostolic Faith religion had increased odds of not using any method of contraception, which is consistent with their beliefs and teachings. Conversely, when SARCs usage is compared to LARCs usage, religion was not found as a predictor, implying religious beliefs or teachings will not influence the choice of contraceptive, but will influence the choice of whether to use or not use contraceptives in general. In Zimbabwe, religion is certainly deep-rooted, and these diverse beliefs and practices have an impact on the choice. Other studies have highlighted the impact of Christianity and, more specifically, the lessened chances of LARCs use, and contraceptives in general, among the Apostolic religion [32].

In addition to the low prevalence of LARC usage, equity concerns remain significant challenges in family planning in Zimbabwe. Similar to findings by Wado and colleagues, 2019 study, women in the poorest and middle wealth classes are less likely to use LARCs compared to those in the highest wealth class [33]. While one might assume that the reason for the low LARC usage among adolescent girls and young women in lower wealth classes was due to greater access to LARC services among those with higher wealth status, this may not have been the case in Zimbabwe. The ASRH program, implemented from 2010 to 2015, specifically aimed to provide nationwide access to LARCs, potentially resulting in more equitable availability across various socio-economic groups [11]. This is evidenced by a study in Ethiopia where subsidized IUDs and implants facilitated equal access to LARCs regardless of socio-economic status services [7]. The underutilization of LARCs among women in poorer wealth classes may be linked to lack of reproductive autonomy, as highlighted in a study by Stonehill and colleagues in 2020. Empowering women is crucial for promoting LARC usage, although conflicting findings exist on the impact of reproductive autonomy among adolescents [7]. Addressing safety concerns, dispelling myths, and enhancing decision-making autonomy among adolescent girls and young women are vital steps to improve LARC utilization and overcome barriers highlighted in various research studies [33].

### 4.1 Strengths and limitations

This cross-sectional study utilized secondary data from the 2015 ZDHS. There are several advantages associated with using ZDHS data, including but not limited to, high participant response rates and wider national coverage [34]. In Zimbabwe, no other national survey gathers health data as extensive as the ZDHS. Furthermore, the ZDHS survey design is subject to international standards with high-quality interviewer training and standardised data collection procedures across 100 countries [34, 35]. This cross-sectional study presents findings that are generalizable and comparable across Zimbabwe.

However, utilizing ZDHS programme data sets also have drawbacks. ZDHS data are gathered through in-person interviews which leaves the data open to social desirability bias, for example, when unmarried adolescent girls are asked whether they are sexually active [7]. Due to the utilization of secondary dataset, the study could not delve into significant contextual factors and country-specific characteristics that might impact LARCs usage in Zimbabwe [17]. Due to the cross-sectional design employed in the study only associative effects could be measured. The cross-sectional design employed in the study cannot make causal or temporal inferences [7, 14].

### 4.2 Future work and recommendations

Future work and recommendations for the study on factors associated with LARC usage in Zimbabwe should focus on addressing disparities between LARC and SARC utilization among adolescent girls and young women. Ideal family planning programs ought to have a balanced mix of contraception methods to satisfy different preferences. It is crucial to ensure a diverse range of contraception methods and services are available to cater to individual preferences effectively. Factors influencing LARC usage such as wealth, religion, parenthood, marital status, and education level should be considered in designing targeted strategies. Additionally, efforts should be directed towards increasing LARC usage among specific groups such as women with primary and secondary education, married women, and members of the Apostolic Faith church. To enhance LARC utilization, it is essential to investigate factors such as knowledge of these methods, availability, accessibility, cost, provider attitudes, previous unplanned pregnancy, exposure to mass media and mHealth, work status, myths and misperceptions related to LARCs and women’s reproductive health decision making autonomy. By addressing these factors and implementing the recommended strategies, there is potential to reduce unintended pregnancies and unsafe abortions among adolescent girls and young women in Zimbabwe.

## 5. Conclusions

Although LARC methods are recommended by WHO, current method use in the study reflects a method mix skewed heavily toward SARCs usage and low LARCs uptake. Several socioeconomic and demographic factors were identified to be important predictors of LARCs usage among Zimbabwean adolescent girls and young women. Acquiring a tertiary education, having living children, a desire to cease bearing children altogether and highest wealth class status were generally associated with increased likelihood of using LARCs. Among other factors being married and being a member of the Apostolic Faith church were associated with a decreased likelihood of LARCs usage. The findings are relevant in the Zimbabwean context and can guide strategies and polices to improve access to and utilization of LARCs in the country. Improved access to and utilisation of LARCs among adolescents and young women helps to improve SRH outcomes such as unfulfilled demand for contraception, adolescent pregnancies, unsafe abortions, avoidable maternal deaths, and preventable neonatal and infant mortality, and give them better educational opportunities and career advancement potential. However, it is also necessary to investigate cultural factors which might also be influencing LARCs usage in Zimbabwe. Most importantly, only barrier methods prevent the transmission of STIs and HIV, therefore, we recommend policies to advocate for dual use of barrier methods and LARCs to effectively prevent unintended pregnancies and provide protection against STIs.

## Supporting information

S1 File The DHS data dictionary for all the independent categorical variables included in the study. (PDF)

S2 File Distribution of contraception methods used by women aged 15–24 in Zimbabwe. (TIF)

S3 File Distribution of contraception method usage grouped by socio-economic and demographic characteristics of sexually active adolescent girls and young women aged 15–24 in ZDHS 2015. (PDF)

S4 File Sexually active adolescent girls and young women aged 15–24 in ZDHS 2015 data set. (XLSX)
