# Supplementary material for: Factors associated with long-acting reversible contraceptives usage among sexually active adolescent girls and young women in Zimbabwe
Source: PLOS Glob Public Health. 2024 Aug 20;4(8):e0003551. doi: 10.1371/journal.pgph.0003551 (PMC11335097; doi:10.1371/journal.pgph.0003551)
Supplement: S1 File — (PDF) [file pgph.0003551.s001.pdf]

| Variable | Categorical variable | Value label |
|---|---|---|
| Age group in 5 years (V013) | 15-19 | 1 |
| | 20-24 | 2 |
| Type of place of Residence (V25) | Urban | 1 |
| | Rural | 2 |
| Highest education levels (V103) | No education | 0 |
| | Primary education | 1 |
| | Secondary education | 2 |
| | Higher education | 3 |
| Religion (V130) | Traditional | 1 |
| | Roman catholic | 2 |
| | Protestant | 3 |
| | Pentecostal | 4 |
| | Apostolic sector | 5 |
| | Other Christian | 6 |
| | Muslim | 7 |
| | None | 8 |
| | Other | 9 |
| Wealth Index (V190) | Poorest | 1 |
| | Poorer | 2 |
| | Middle | 3 |
| | Richer | 4 |
| | Richest | 5 |
| Number of living children (V218) | | |
| Current marital status (V501) | Never in union | 0 |
| | Married | 1 |
| | Living with partner | 2 |
| | Widowed | 3 |
| | Divorced | 4 |
| | No longer living together | 5 |
| Desire for children in the future | Wants within in 2 years | |
| | Wants after 2+ years | |
| | Wants, unsure timing | |
| | Undecided | |
| | Wants no more | |
| | Sterilized (respondent or partner) | |
| | Declared infecund | |
| | Never had sex | |
