# Supplementary figures and images for: Factors associated with long-acting reversible contraceptives usage among sexually active adolescent girls and young women in Zimbabwe
Source: PLOS Glob Public Health. 2024 Aug 20;4(8):e0003551. doi: 10.1371/journal.pgph.0003551 (PMC11335097; doi:10.1371/journal.pgph.0003551)

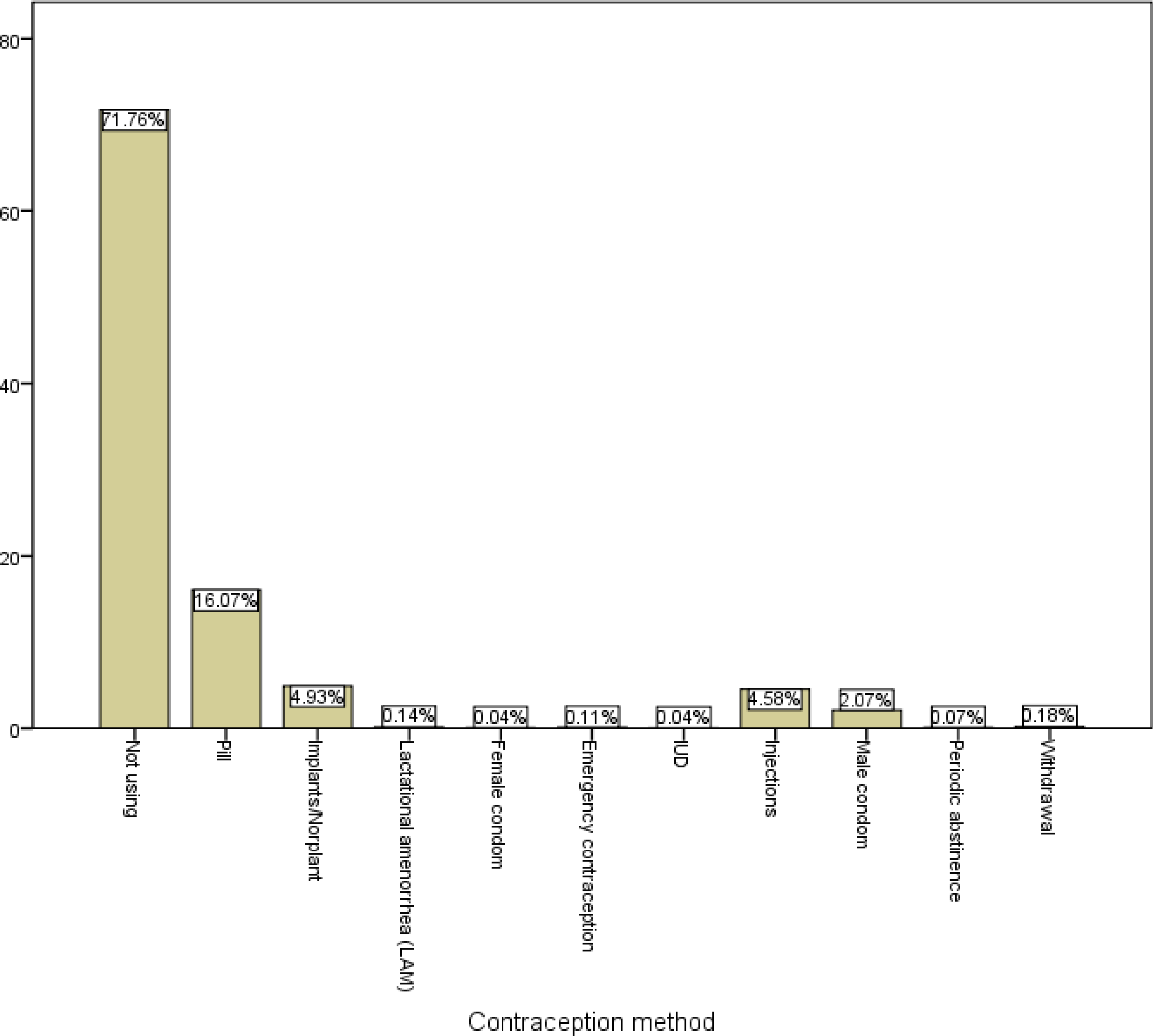

Supplement: S2 File — (TIF) [file pgph.0003551.s002.tif]
