# Supplementary material for: Factors associated with long-acting reversible contraceptives usage among sexually active adolescent girls and young women in Zimbabwe
Source: PLOS Glob Public Health. 2024 Aug 20;4(8):e0003551. doi: 10.1371/journal.pgph.0003551 (PMC11335097; doi:10.1371/journal.pgph.0003551)
Supplement: S3 File — (PDF) [file pgph.0003551.s003.pdf]

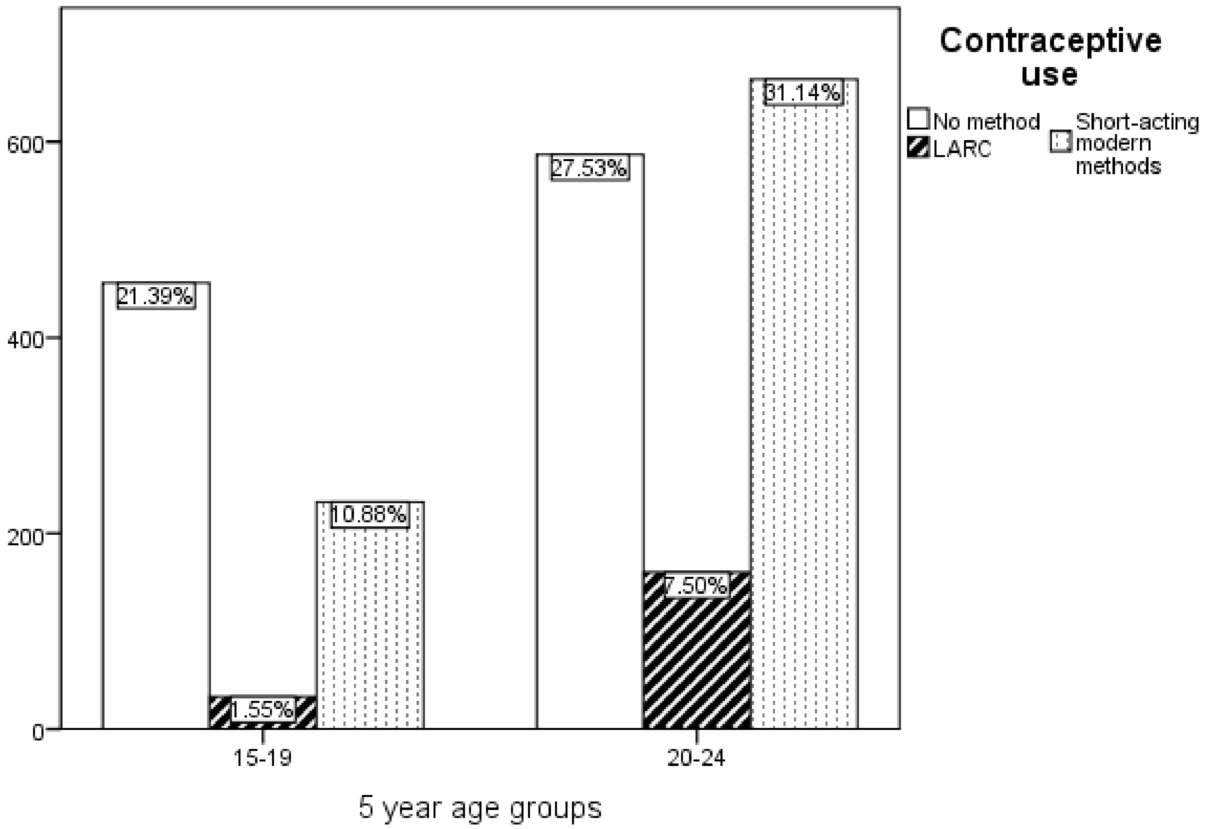


**Fig 1:** Distribution of contraception method usage grouped by age groups.

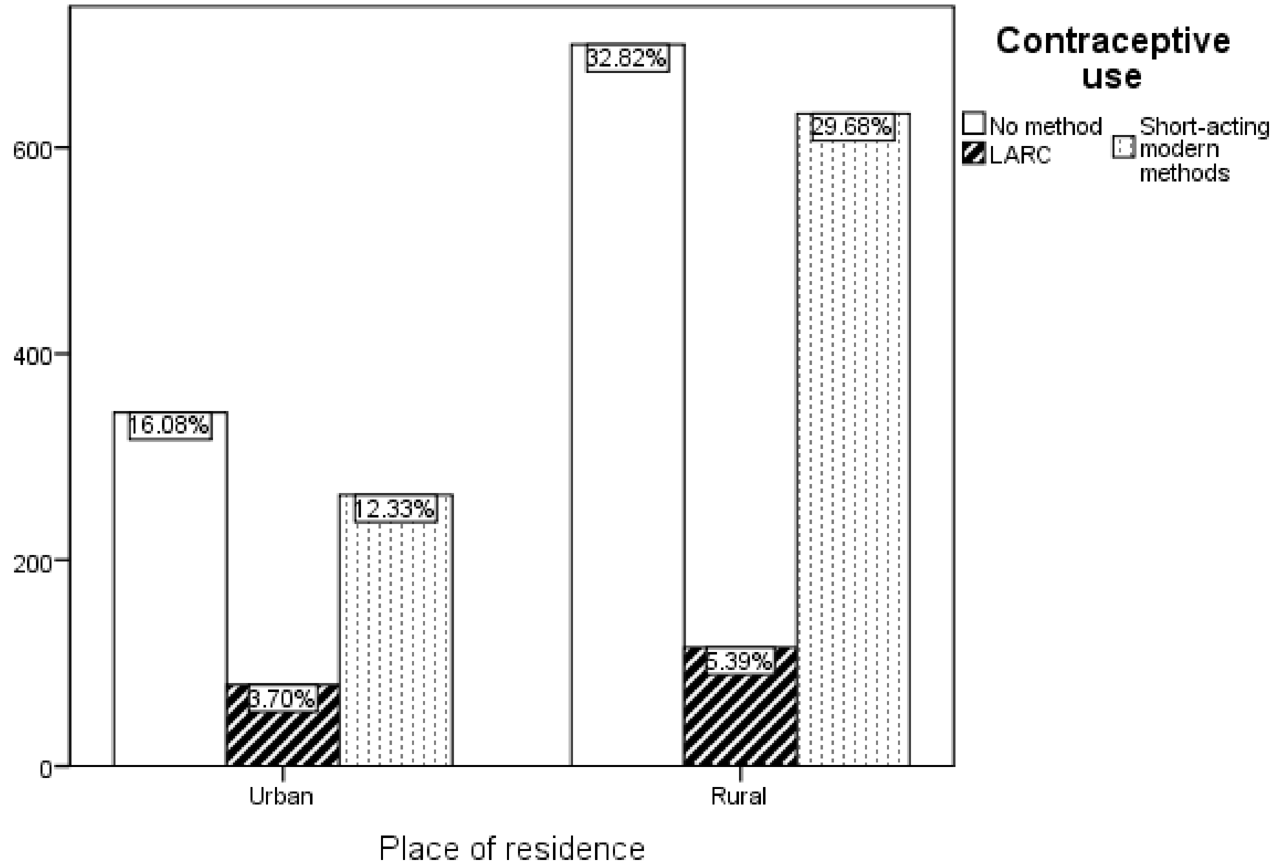

**Fig 2:** Distribution of contraception method usage grouped by place of residence.

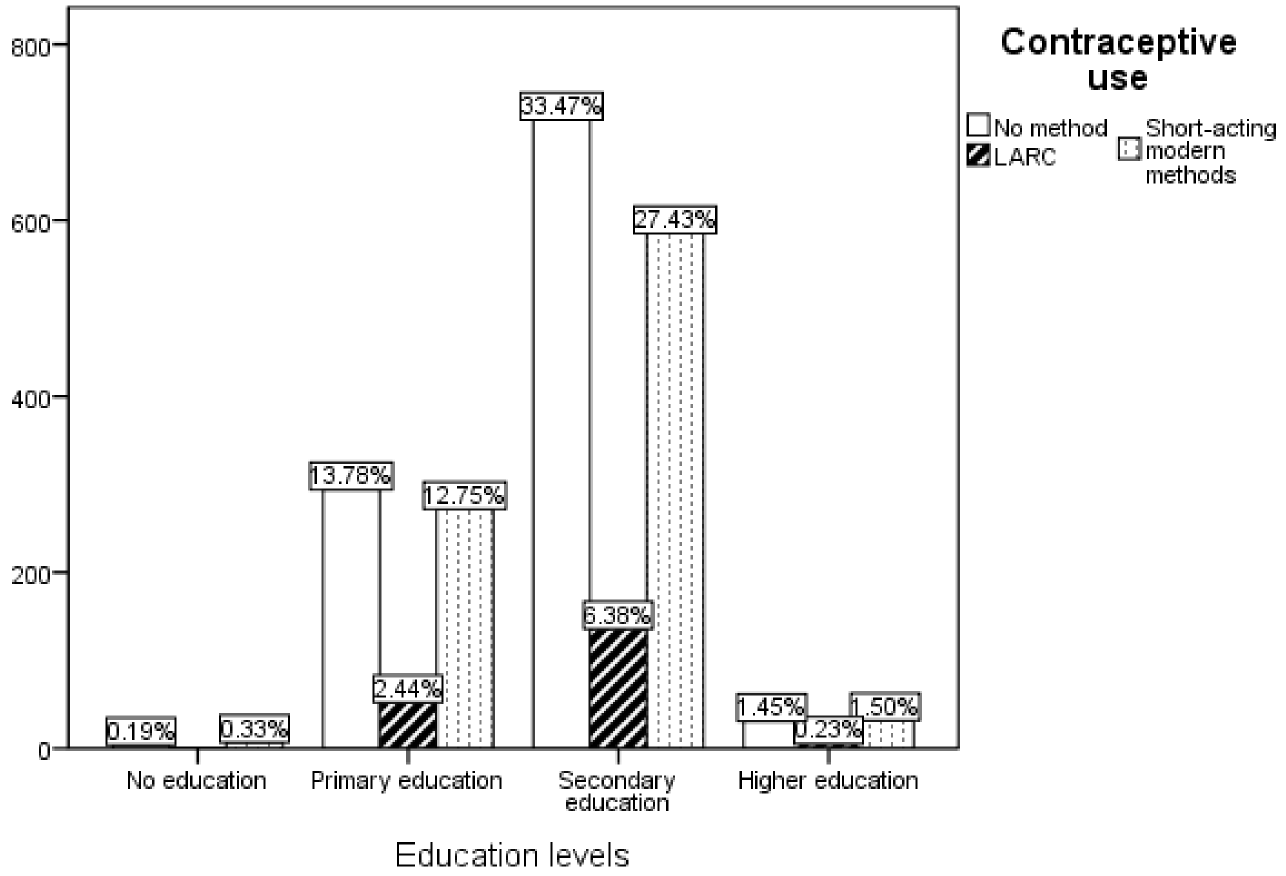


**Fig 3:** Distribution of contraception method usage grouped by highest education level.

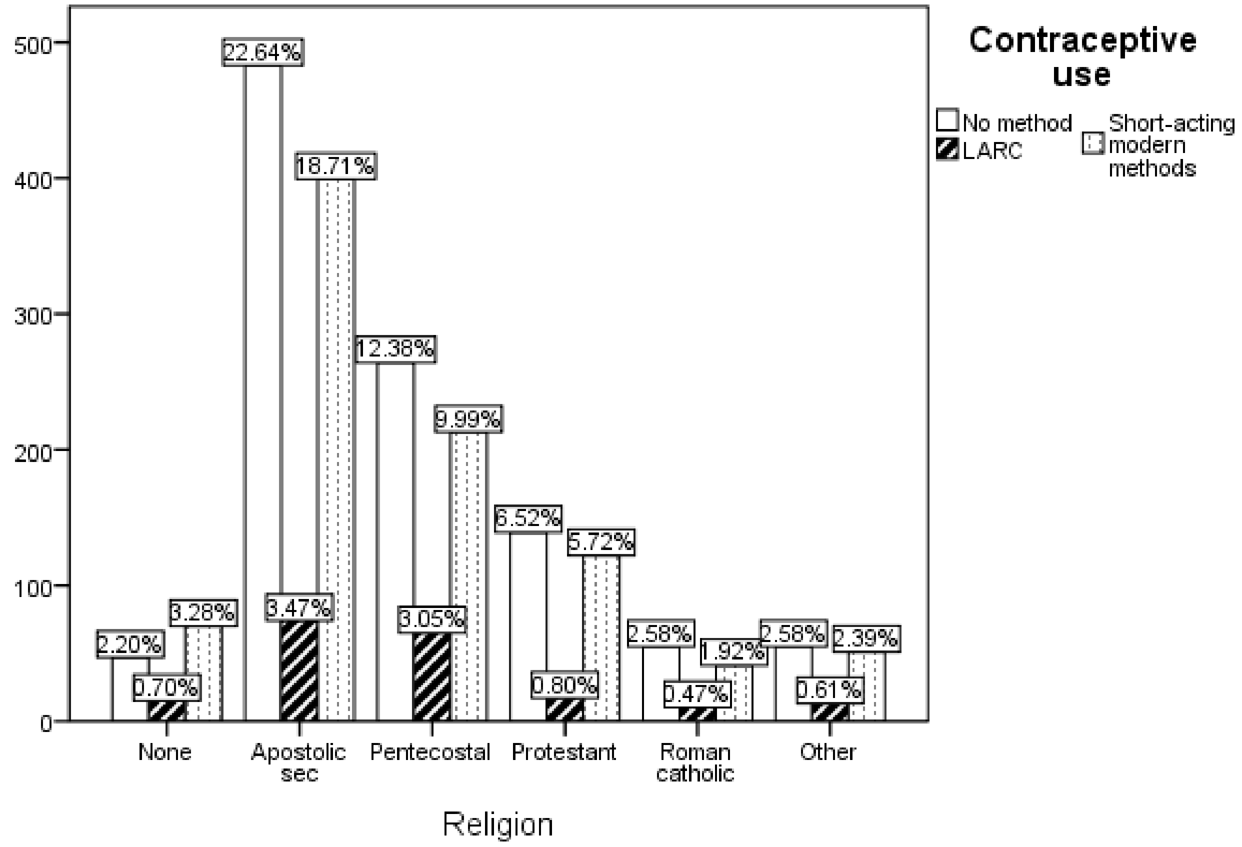


**Fig 4:** Distribution of contraception method usage grouped by highest education level.
by religion.

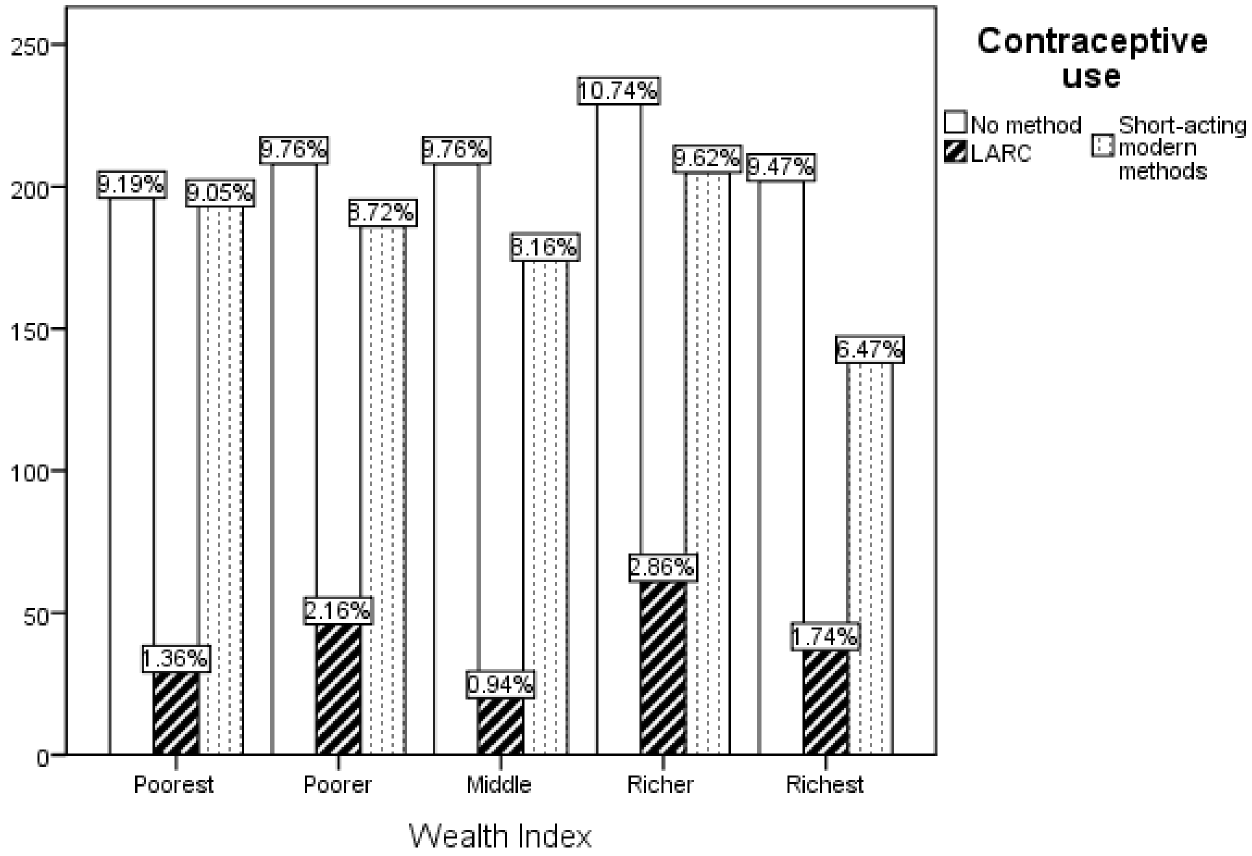


**Fig 5:** Distribution of contraception method usage grouped by highest education level. by wealth status.

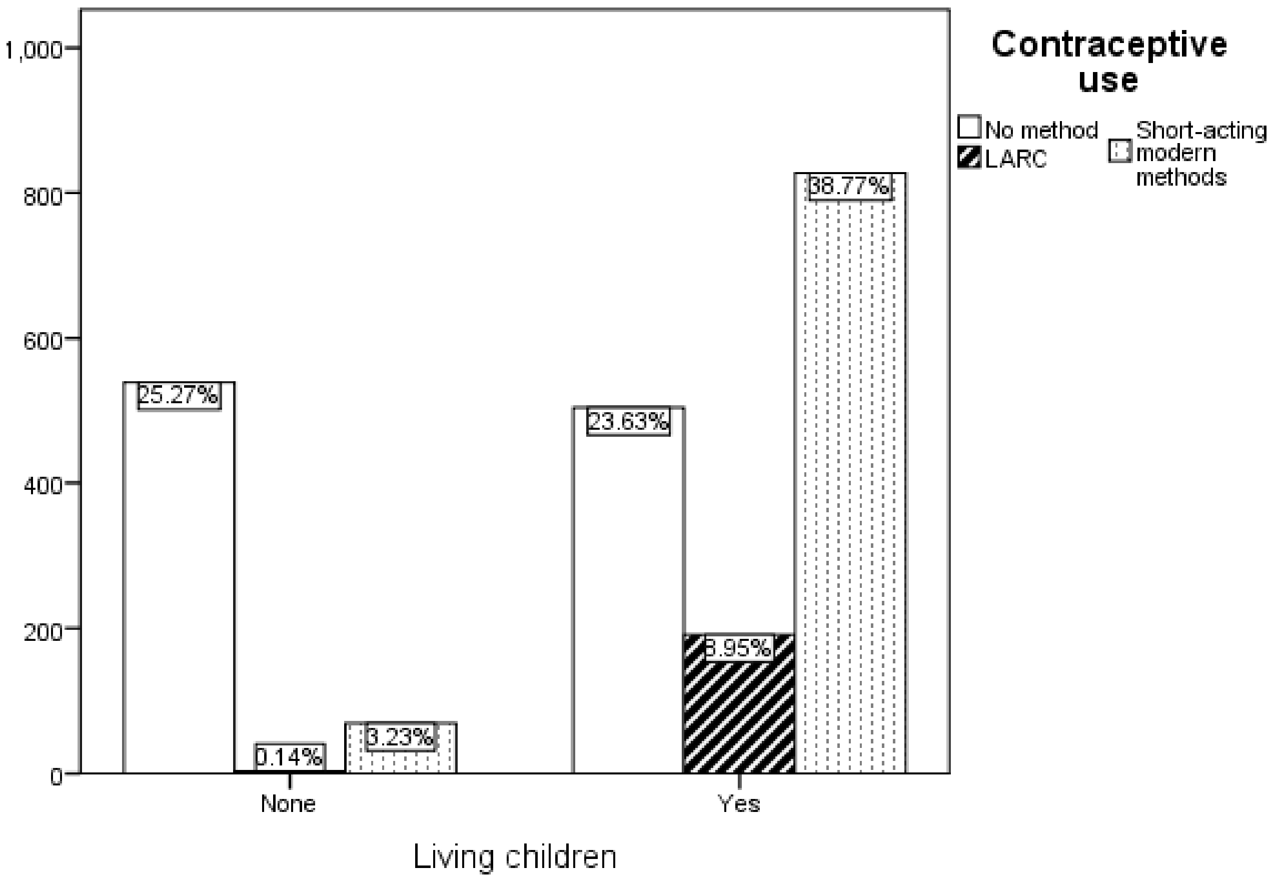


**Fig 6:** Distribution of contraception method usage grouped by whether a woman has living children not.

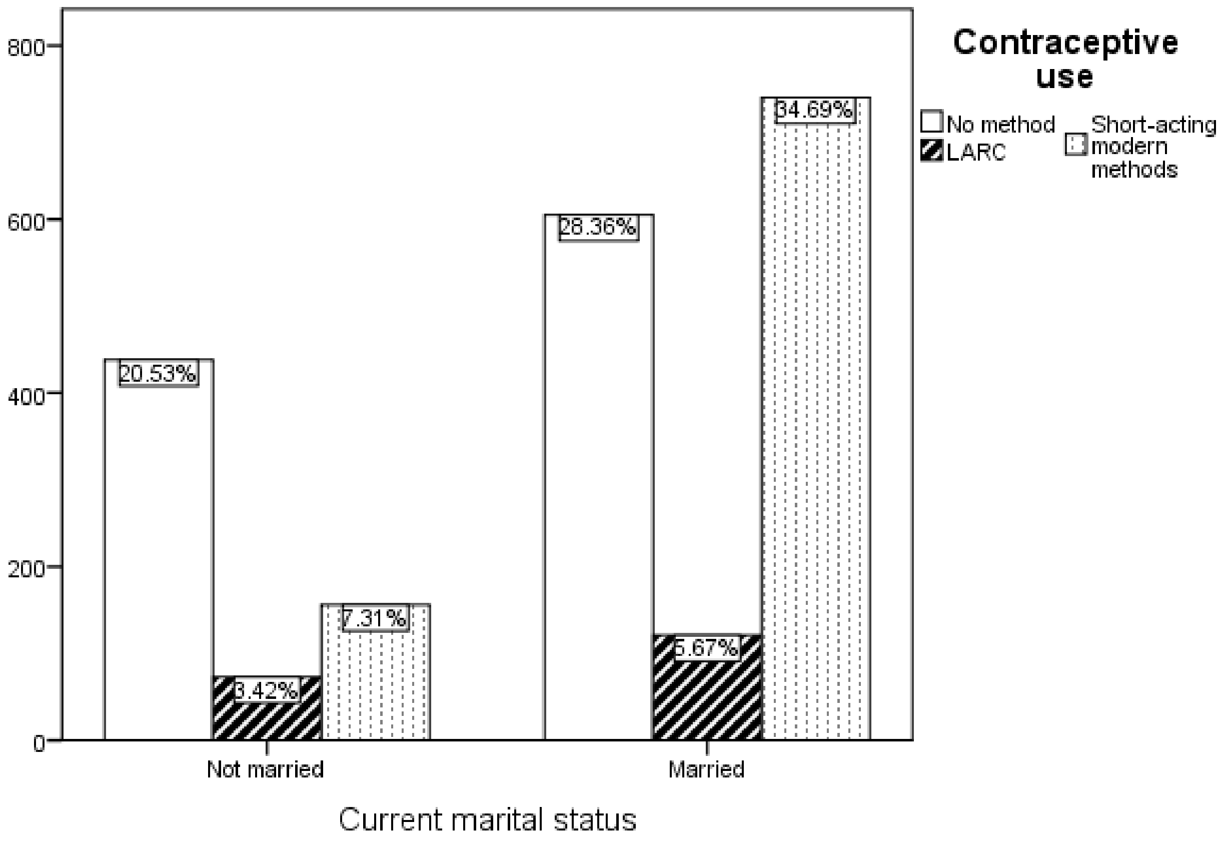


**Fig 7:** Distribution of contraception method usage grouped by current marital status.

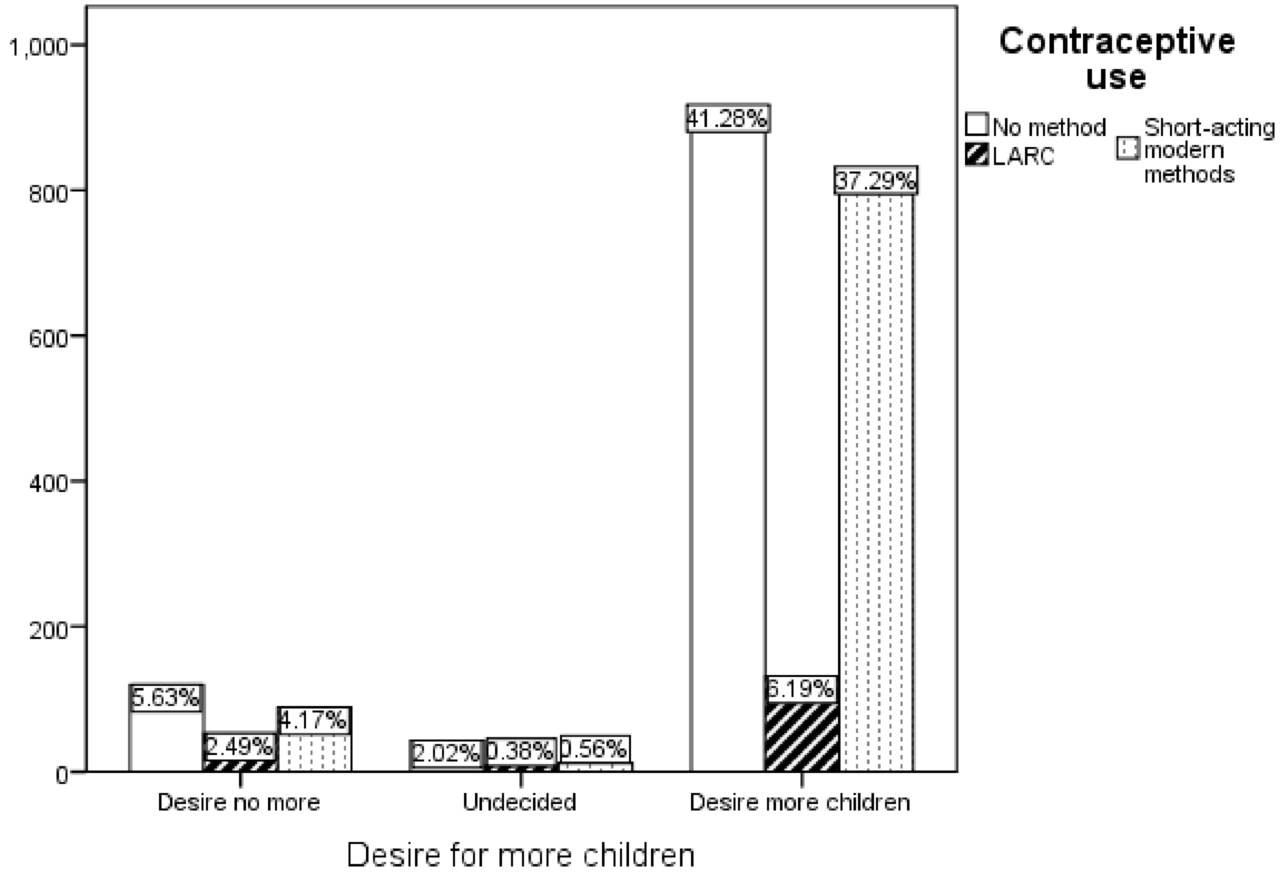


**Fig 8:** Distribution of contraception method usage by highest education level. grouped by desire for more children.
